# Aneuploidy during development in facultative parthenogenetic *Drosophila*

**DOI:** 10.1038/s41437-023-00664-z

**Published:** 2023-11-28

**Authors:** A. L. Sperling, D. M. Glover

**Affiliations:** 1 Department of Genetics, University of Cambridge, Cambridge, UK; 2 Division of Biology and Biological Engineering, California Institute of Technology, Pasadena, CA USA

**Keywords:** Development, Evolutionary genetics

## Abstract

From concatenated chromosomes to polyploidization, large-scale genome changes are known to occur in parthenogenetic animals. Here, we report mosaic aneuploidy in larval brains of facultatively parthenogenetic *Drosophila*. We identified a background of aneuploidy in *D. mercatorum* strains and found increased levels of aneuploidy in the larval brain tissue of animals arising parthenogenetically versus those arising from sexual reproduction. There is also intra-individual variation in germline-derived aneuploidy within the same strain. To determine if this is a general feature of facultative parthenogenesis in drosophilids, we compared sexually reproduced and parthenogenetic offspring from an engineered facultative parthenogenetic strain of *D. melanogaster*. In addition to germline-derived aneuploidy, this revealed somatic aneuploidy that increased by up to fourfold in parthenogens compared to sexually reproduced offspring. Therefore, the genetic combination identified in *D. mercatorum* that causes facultative parthenogenesis in *D. melanogaster* results in aneuploidy, which indicates that the loss of mitotic control resulting in parthenogenesis causes subsequent genome variation within the parthenogenetic offspring. Our findings challenge the assumption that parthenogenetic offspring are near genetic replicas of their mothers.

## Introduction

Parthenogenesis, first observed in aphids by Charles Bonnet in the 1740s, requires the initiation of embryonic development in unfertilized eggs and manifests in two primary forms: obligate, where animals reproduce almost entirely asexually, and facultative, where animals have the ability to alternate between sexual and asexual reproduction (reviewed in Engelstadter 2008; Markow 2013; Sperling and Glover 2023a; Suomalainen 1950). Some animals appear to reproduce asexually by skipping meiosis completely, undergoing a mitotic-like cell division termed *apomixis*, whereas most parthenogenetic animals likely undergo *automixis*, in which meiosis is fully or partially completed. In parthenogenetic drosophilids, meiosis proceeds to completion creating the four haploid products (one pronucleus and three polar bodies) and rediploidization is achieved by combining the haploid products of meiosis or through whole genome duplication (Sperling and Glover 2023a). Subsequently, centrosomes, which are microtubule organizing centers (MTOCs) required for fidelity of the embryonic nuclear divisions (Basto et al. 2006), form de novo and mitosis is initiated (Sperling et al. 2023). In *Drosophila* facultative parthenogens, some misregulation of mitosis allows for the initiation of parthenogenesis and, as a consequence of this diminished control, leads to disorganized cell divisions, DNA segregation errors, and the generation of non-diploid nuclei in early embryos (Eisman and Kaufman 2007; Sperling et al. 2023). Despite this, fertile, viable animals still result. This raises a paradox: it is necessary to inactivate regulatory elements of mitosis for parthenogenesis to be initiated but mitotic regulation is necessary for the animal to develop correctly. If or how control over the cell cycle is regained after the initiation of parthenogenesis is an important question regarding offspring fitness and thus evolution of parthenogenetic reproduction in *Drosophila* and likely other parthenogenetic animals. There has been no study of whether control over mitosis is regained during development.

When the initiation of mitosis is not precisely regulated, it can lead to genome and chromosome instability, with the consequence of aneuploidy. Aneuploidy is characterized by intra- or inter-individual deviation of the whole or partial chromosome complement from the population norm. Aneuploidy is primarily associated with age-related disease progression, although it has also been observed in certain healthy tissues such as mouse neuroblasts or hepatocytes (Rehen et al. 2001; Rehen et al. 2005; Santaguida and Amon 2015; Siegel and Amon 2012; Yurov et al. 2005; Yurov et al. 2007). Four primary causes of aneuploidy have been described (reviewed in Sansregret and Swanton 2017; Siegel and Amon 2012): defects in the mitotic checkpoint; defects in microtubule attachment; centrosome amplification; and chromosome adhesion defects. Furthermore, aneuploidy is thought to further exacerbate genome and chromosome instability by inducing DNA damage (Santaguida and Amon 2015). While aneuploidy is rare outside of documented diseases such as cancer, it has been observed in several animal species, including certain parthenogenetic dipteran species that undergo somatic chromosome or sex chromosome elimination (Sperling and Glover 2023a). In facultative parthenogenetic *Drosophila*, there have been no documented instances of aneuploidy, although mosaicism and polyploidy have been observed (Sperling et al. 2023; Stalker 1954). Thus, there is a complex but poorly understood interplay between parthenogenesis, polyploidy, and genome and chromosome stability.

The initiation of the nuclear division cycle in unfertilized *Drosophila* eggs during facultative parthenogenesis has revealed notable changes in centrosome function together with cell growth and metabolism (Sperling et al. 2023). A genetic basis for this phenomenon has been attributed to increased expression of the proto-oncogenes *Myc* and the mitotic protein kinase *polo*, coupled with decreased expression of the metabolic gene *Desat2*. Collectively, these gene alterations drive the occurrence of facultative parthenogenesis in non-parthenogenetic *D. melanogaster*. As a consequence, the resulting offspring exhibit varying ploidy levels, yielding diploid, triploid, and tetraploid individuals, among which triploids predominate. The proto-oncogene transcription factor Myc has a conserved role in *Drosophila* (Grifoni and Bellosta 2015) and is known to induce aneuploidy when overexpressed in human cancer (Dang 2012; Sansregret and Swanton 2017). During parthenogenesis, Myc prepares the egg for initiating development and the resulting embryo for continuing development. The mitotic protein kinase Polo, Plk1 in mammalian cells, is often upregulated in tumor cells (Takai et al. 2005), and during parthenogenesis in *D. melanogaster* Polo contributed to centrosome formation. *Desat2* encodes a desaturase involved in lipid metabolism which contributes to cold tolerance when its expression is reduced (Greenberg et al. 2003), and during parthenogenesis it facilitates positioning of the polar bodies proximal to the female pronucleus thereby enabling di-, tri-, and tetraploidization. Prior to the identification of genetic factors underlying parthenogenesis in *Drosophila*, chromosome or genome instability had already been observed in the developing parthenogenetic embryos of *D. mercatorum* (Eisman and Kaufman 2007). Together, these findings illustrate the intricate molecular mechanisms underlying facultative parthenogenesis and the connection between dysregulation of cell division, chromosome and genome instability, and the initiation of asexual reproduction.

In this study, we aimed to determine how the aneuploidy that we observed in many *D. mercatorum* strains was connected to parthenogenesis. Remarkably, we observed the presence of both germline-derived aneuploidy and somatic aneuploidy in both sexually reproducing and parthenogenetic *D. mercatorum*, a finding that was replicated in genetically engineered sexually reproducing and parthenogenetic *D. melanogaster*. These findings strongly support the notion that aneuploidy arises due to the genetic alterations that enable parthenogenesis in *Drosophila*. Such abnormalities are propagated or retained within the tissues of facultative parthenogenetic offspring. Furthermore, our results imply that sexual reproduction maintains the integrity of the genome, even in less favorable genetic backgrounds. By contrast, facultative parthenogenesis leads to elevated levels of intra-individual genomic variability, driving genetic diversity in the offspring. This highlights the critical role of sexual reproduction in preserving genome fidelity and suggests that facultative parthenogenesis contributes to increased genomic instability within individuals and their progeny.

## Materials and methods

### Drosophila stocks

The *Drosophila mercatorum* stocks used in this study: the sexually reproducing *D. mercatorum* from Praia Grande, Sao Paulo, Brazil, Cornell Stock Centre: 15082–1511.00, named Sexually Reproducing 1. The sexually reproducing *D. mercatorum* from Kamuela, Hawaii, USA, Cornell Stock Centre: 15082–1521.22, named Sexually Reproducing 2. Facultative parthenogenetic *D. mercatorum*, Cornell Stock Centre: 15082–1527.01, named Facultative 1. Facultative parthenogenetic *D. mercatorum*, Cornell Stock Centre: 15082–1527.02, named Facultative 2. Facultative parthenogenetic *D. mercatorum*, Cornell Stock Centre: 15082–1527.03, named Facultative 3. Facultative parthenogenetic *D. mercatorum*, Cornell Stock Centre: 15082–1527.05, named Facultative 5. Parthenogenetic *D. mercatorum*, Hawaii Highway km 28. Hawaii, Cornell Stock Centre: 15082–1525.07. The *Drosophila melanogaster* stocks used in this study were CB1 a wild caught strain from Cambridge, UK, *Desat2[7-11HD-low]* (Bloomington *Drosophila* Stock Center: 4532), *Desat2, Desat1[ey07679]* (Bloomington *Drosophila* Stock Center: 20171), *Myc[Dp(1;3)DC059]* (Bloomington *Drosophila* Stock Center: 31438), and *GFP-polo[+]* (Claudio Sunkel Lab).

### Mitotic chromosome preparation

Brains were dissected from 3rd instar larvae in saline (0.7%NaCl) and cultured for 1.5 h in 10 μM colchicine diluted in 0.7% NaCl. The brains were then subjected to hypotonic shock by incubation in 0.5% trisodium citrate for 9 min. The brains were then fixed by a 60 s incubation in 45% acetic acid followed by 5 min in 60% acetic acid on a coverslip. A slide was placed over the coverslip and squashed between two sheets of blotting paper. Immediately after squashing, the preparation was frozen in liquid Nitrogen and the coverslip removed using a scalpel. The squashed brain preparation was then dehydrated by successive 5 min incubations in 70% and 100% ethanol and then air-dried. If in situs were performed, slides were baked at 58 °C for 1 h in a dry oven. The preparations were then denatured with 70% formamide in 2 × SSC at 70 °C for 20 min and subjected to further dehydration by successive 5 min incubations in 70% and 100% ethanol, air-drying and immediate application of the HRC protocol.

### Tissue fixation

Brains were dissected in 0.2% PBT (PBS + 0.2% Tween), fixed for 20 min in 4% paraformaldehyde/PBT, washed with PBT, blocked with PBT + 10% BSA for 1 h, and incubated with the primary antibody in PBS + 2% Tween + 1% BSA for 16–24 h at 4 °C. After washing the brains with PBT three times for a total of 25 min, they were incubated with the secondary antibody for 2 h at RT, or 16 h at 4 °C. Phalloidin staining for visualizing F-actin was done for 20 min, either after fixing or after secondary antibody staining. Finally, the ovaries were washed twice with PBT for a total of 40 min and mounted in Vectashield (Vector) with DAPI for visualizing DNA. Unless specified, all steps were performed at room temperature.

#### Primary antibodies

Mouse α-Tubulin antibody, DM1A (1:200 dilution), rabbit α-Cnn antibody, Glover Lab (1:200 dilution), guinea pig α-Asterless antibody, a kind gift from the Nasser Rusan Lab (1:50,000).

#### Secondary antibodies

(All 1:500 dilutions) Goat α-Mouse 488 and 647 from Life Technologies, Goat α-Rabbit 488 from Invitrogen and 647 from Life Technologies. Goat α- guinea pig 647 from Life Technologies.

### Molecular instruments HCR in situ sample preparation

All materials including buffers and probes were purchased or gifted from Molecular Instruments. To ensure optimal hybridization to mitotic chromosome preparations, probes were selected for accessible gene regions by the criteria that the chosen genes were highly transcribed in brain tissue. The standard Molecular Instruments HCR protocol (v3.0) for sample on slide was used.

### Imaging

All images were acquired on a Leica SP8 confocal microscope, and the images were minimally optimized for brightness and contrast using ImageJ (Schindelin et al. 2012). No other image alteration was performed. All images presented are projections of multiple focal planes.

### Statistical analysis

Fisher’s Exact Test (in R) was used to determine if the changed in aneuploidy were significant. It was chosen because it is permissive to having samples with low instances of positive cases. The Pearson’s correlation coefficient was calculated in Excel and the linear regression analysis was also performed in Excel.

## Results

**Fig. 1:**
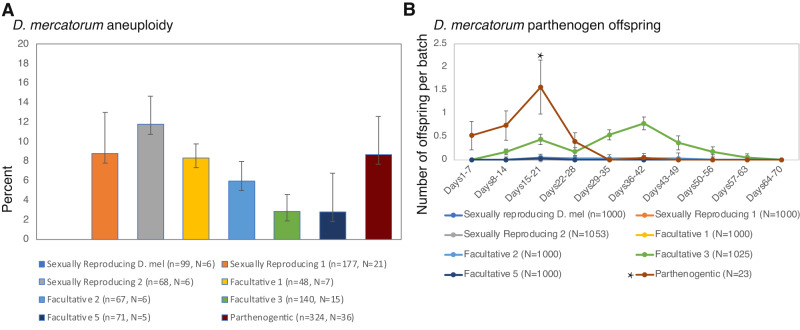
*D. mercatorum* karyotypes and parthenogenetic ability. **A** Percentage of cells in *D. mercatorum* 3rd instar larvae brain tissue with aneuploidy, with a *D. melanogaster* control. The Pearson’s correlation coefficient (*r*) was −0.53 and the significance (*p*) was 0.44. Number of cells screened, (*n*); number of animals in which the cells were screened (*N*). **B** Parthenogenetically produced adult offspring per batch of 25 females versus maternal age for *D. mercatorum* and a *D. melanogaster* control. Parthenogenetically produced embryos, larvae, or pupae that died before reaching adulthood are excluded. The total number of female mothers screened is given. *This is a fully parthenogenetic strain that was tested in batches of 1 and thus fewer females were tested. The names attributed to the strains were taken from their designation by the Cornell National Species Stock Center. Error bars represent standard error.

We first sought to gain an indication of the frequency by which aneuploidy arises in sexually reproducing and parthenogenetic strains of *D. mercatorum*. We noted aneuploidy within neuronal tissue of a parthenogenetic strain as well as six sexually reproducing strains, four of which that showed a higher degree of facultative parthenogenesis are henceforth named Facultative. We observed that 2.8–11.8% of cells had a loss or a gain of 1 or more chromosomes (Fig. 1A). There is no observed aneuploidy in a wild-type strain of sexually reproducing *Drosophila* (CB1, wild-caught in 2019). When we compared the incidence of aneuploidy to previously documented parthenogenetic ability (Fig. 1B) (Sperling et al. 2023), we found no clear correlation between parthenogenesis and aneuploidy in the different *D. mercatorum* strains (*r* = −0.35, *p* = 0.44). In wildtype *D. melanogaster* there was also no adult parthenogenetic offspring produced. All the sexually reproducing *D. mercatorum* strains examined were capable of different degrees of facultative parthenogenesis, some producing only embryos and others producing adult flies (Sperling et al. 2023; Sperling and Glover 2023b). We found that the completely parthenogenetic strain displayed 8.7% of cells with aneuploidy whereas the sexually reproducing strain with the highest level of facultative parthenogenesis showed only 2.8% aneuploidy. Therefore, the level of facultative parthenogenesis may not be an indicator of the prevalence of aneuploidy in the larval brain, but it may be a consequence of the underlying cause of parthenogenesis. It therefore seemed possible that a connection between parthenogenesis and aneuploidy might only be apparent when comparing parthenogens to sexually reproduced females from the same strain.

**Fig. 2:**
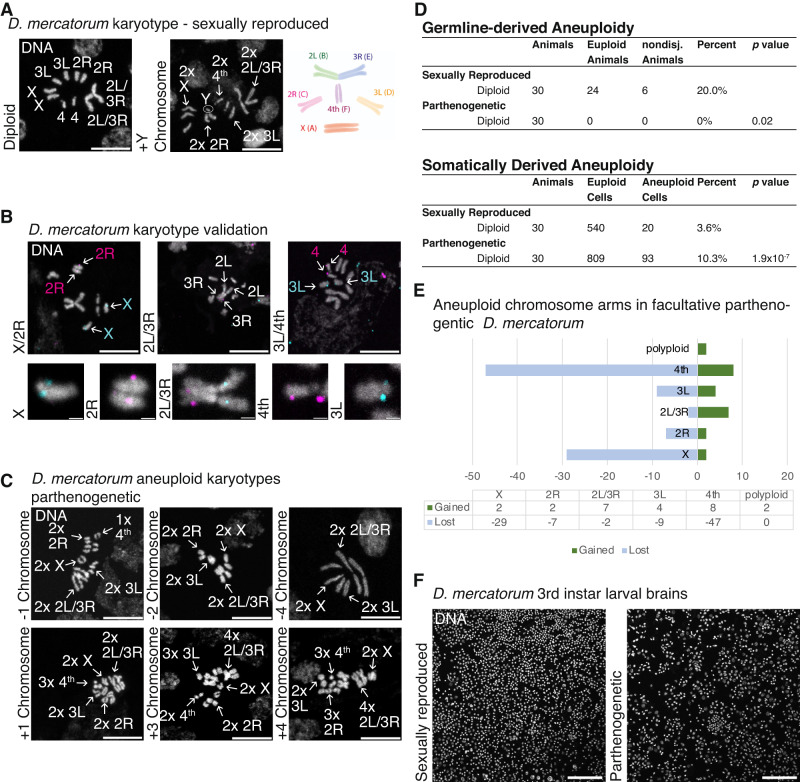
Sexually reproducing and parthenogenetic *D. mercatorum* karyotypes and aneuploidy. **A** Sexually reproduced *D. mercatorum* 3rd instar larvae brain tissue karyotype. DAPI/DNA (white). Scale bar, 1 μm. **B** Parthenogenetically reproduced *D. mercatorum* 3rd instar larvae brain tissue karyotypes, the offspring were all diploid. **C** Examples of aneuploid karyotypes from the parthenogenetically reproduced *D. mercatorum* 3rd instar larvae brain tissue. Examples include the loss of 1, 2, or 4 chromosomes and the gain of 1, 3 or 4 chromosomes. **D** Table of germline-derived and somatically derived aneuploidy, including offspring ploidy, number of animals examined, euploid cells, aneuploid cells, percent, and *p* value. Those that were counted of germline aneuploid were not counted towards the somatically derived aneuploidy, unless they had additional chromosome changes. The *p* value was calculated with Fisher’s exact test. **E** Chart of the parthenogenetic brain chromosome gains and losses by chromosome arm. **F** Sexually reproduced and parthenogenetic *D. mercatorum* 3rd instar larvae brain tissue. Nuclei in cells from parthenogenetic brains appear larger despite both being diploid. DAPI/DNA (white). **A**–**C** Scale bars 10 μm, B 1 μm and **F** 100 μm.

We proceeded to investigate the larval brain tissue of both sexually reproduced and parthenogenetic *D. mercatorum* offspring from a strain that exhibited an average of one adult progeny per ten virgin females screened (equivalent to 10.7% offspring per total number of females screened) (Fig. 1B, Facultative 3) (Sperling et al. 2023). The karyotype of *D. mercatorum* comprises the X chromosome (corresponding to Muller element A); the 2 L chromosome arm (Muller element B) fused to the 3 R arm (Muller element E); a telocentric 2 R arm (Muller element C); a telocentric 3 L arm (Muller element D) which is slightly larger than the telocentric 2 R arm; and the 4th chromosome (Muller element F) (Fig. 2A) (DeSalle et al. 1986; Sperling et al. 2023). The Muller elements terminology reflects the conservation of chromosome arm content in *Drosophila* species (Ashburner 1989; Muller 1940; Schaeffer 2018; Whiting et al. 1989). *D. mercatorum* shows chromosome polymorphisms for the 4th chromosome (Muller element F) and inversions present on the 2 L chromosome arm (Muller element B) (Sperling et al. 2023). The karyotype of facultative parthenogenic *D. mercatorum* was the same as previously published (Sperling et al. 2023). However, three of the sexually reproduced female-appearing offspring of *D. mercatorum* were mosaic for the presence of one or more Y chromosomes in addition to two X chromosomes, representing a germline derived aneuploidy (Fig. 2A). In *D. mercatorum* the Y chromosome is exceptionally small (Fig. 2A) (DeSalle et al. 1986). Due to the karyotype polymorphisms, we first verified the chromosome arms of the facultative parthenogen matched either the previously published karyotypes of the sexually reproducing and parthenogenetic *D. mercatorum* (Sperling et al. 2023). Using a fluorescence in situ hybridization chain reaction (HCR) protocol adapted for DNA sequence localization (Sperling et al. 2023), we unambiguously identified the individual chromosome arms in the facultative strain of *D. mercatorum* (Fig. 2B). This enabled us to identify which chromosome arms contribute to the aneuploidy phenotype. We were also able to classify the karyotype as matching that of the parthenogenetic *D. mercatorum* for the 4th chromosome polymorphism.

We observed that while both sexually reproduced and parthenogenetic offspring derived from the same lineage were diploid, 10.3% of the mitotic cells in 3rd instar larval brains of parthenogens were aneuploid in contrast to 3.6% in sexually reproduced offspring (Fig. 2C, D and Table S1A). In the parthenogenetic offspring, losses of chromosome arms were more prevalent than gains (Fig. 2E). The greater frequency of losses than gains has also been reported in neuroblasts and hepatocytes of mice (Rehen et al. 2001; Yurov et al. 2007). The chromosomes most frequently were lost in *D. mercatorum* were the X (*n* = 29) and 4th (*n* = 47) chromosome arms (Fig. 2E). The mitotic cells were not clustered within the tissue and the incidence of aneuploidy was evenly distributed (Fig. 2F). The nuclei in the tissue of the parthenogenetic offspring were less densely packed indicating the cells are larger compared to the sexually reproducing offspring. Therefore, parthenogenesis correlates with both aneuploidy and morphological changes to the tissue.

**Fig. 3:**
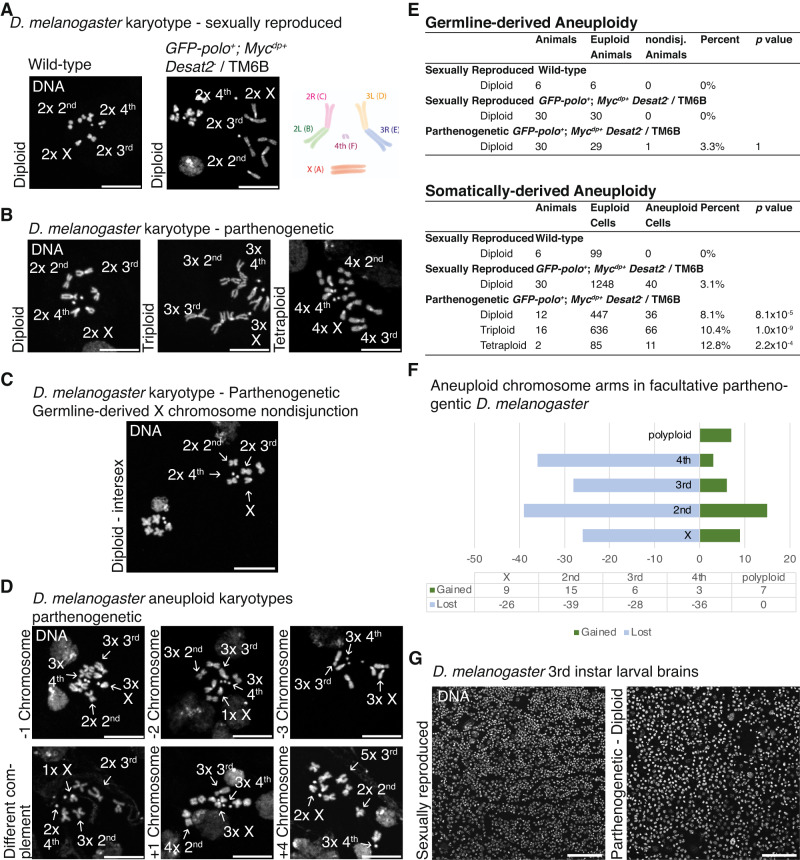
Sexually reproducing and parthenogenetic *D. melanogaster* karyotypes and aneuploidy. **A** Sexually reproduced *GFP-polo*^*+*^*; Myc*^*dp+*^ *Desat2*^*-*^/TM6B 3rd instar larvae brain tissue karyotype. **B** Parthenogenetically reproduced *GFP-polo*^*+*^*; Myc*^*dp+*^ *Desat2*^*-*^/TM6B 3rd instar larvae brain tissue karyotypes, the offspring were diploid, triploid, or tetraploid. **C** Parthenogenetically reproduced *GFP-polo*^*+*^*; Myc*^*dp+*^ *Desat2*^*-*^/TM6B 3rd instar larvae brain tissue with germline-derived X chromosome nondisjunction resulting in aneuploidy. **D** Examples of aneuploid karyotypes from the parthenogenetically reproduced *GFP-polo*^*+*^*; Myc*^*dp+*^ *Desat2*^*-*^/TM6B 3rd instar larvae brain tissue. Examples include the loss of 1–3 chromosomes; a different chromosome complement; and the gain of 1 or 4 chromosomes. **E** Table of germline-derived and somatically derived aneuploidy, including offspring ploidy, number of animals examined, euploid cells, aneuploid cells, percent, and *p* value. Those that were counted of germline aneuploid were not counted towards the somatically derived aneuploidy, unless they had additional chromosome changes. The *p* value was calculated with Fisher’s exact test between the sexually reproducing and the parthenogenetic. **F** Chart of the chromosome gains and losses in the parthenogenetic offspring by chromosome. **G** Sexually reproduced and parthenogenetic *GFP-polo*^*+*^*; Myc*^*dp+*^ *Desat2*^*-*^/TM6B 3rd instar larvae brain tissue. The parthenogenetic nuclei appear larger despite both being diploid. DAPI/DNA (white). **A**–**D** Scale bar, 10 μm and **G** Scale bar,100 μm.

We previously identified genotypes able to confer facultative parthenogenetic ability to *D. melanogaster* by mimicking transcriptomic differences between parthenogenetic and sexually reproducing strains of *D. mercatorum*. We therefore asked whether we could detect aneuploidy in this genetically induced facultative parthenogenetic strain of *D. melanogaster*. *D. melanogaster* diverged from *D. mercatorum* approximately 47 million years ago (Suvorov et al. 2022), and thus its genome architecture is substantially different. *D. melanogaster*’s karyotype includes the X chromosome (Muller element A); chromosome 2, comprising a 2 L arm (Muller element B) and a 2R arm (Muller element C); chromosome 3, consisting of a 3 L arm (Muller element D) and a 3R arm (Muller element E); and the 4th chromosome (Muller element F). In the wild-type D. melanogaster, none of the 99 cells in the 6 animals examined displayed aneuploidy (Figs. 1A, 3A, E). Therefore, unlike *D. mercatorum*, aneuploidy in *D. melanogaster* is not a common feature.

We next examined sexually reproducing *D. melanogaster* that are able to generate 1.4% offspring by facultative parthenogenesis when sexually isolated. The maternal genotype (*GFP-polo*^*+*^; *Myc*^*+dp*^ *Desat2*^*-*^/TM6B) consists of two extra copies of *polo* as a GFP-tagged transgene, one extra copy of *Myc* as a genome duplication of the locus translocated on the 3rd chromosome (endogenous *Myc* is on the X chromosome), and a 16 bp deletion in the 5′ UTR of *Desat2* causing decreased expression (Takahashi et al. 2001). None of the sexually reproduced animals had germline-derived aneuploidy. However, in the larval brain tissue of sexually reproduced *GFP-polo*^*+*^; *Myc*^*+dp*^ *Desat2*^*-*^/TM6B larvae, we identified 3.1% of mitotic cells having somatically derived aneuploidy (Fig. 3A, E). These sexually reproduced animals were exclusively diploid, in contrast to the facultative parthenogenetically produced offspring, which exhibited diploid, triploid, and tetraploid karyotypes (Fig. 3B, E). We observed aneuploidy in up to 40.0% of larval brain cells of the parthenogen offspring (Fig. 3D, E and Table S1B). The average percentage of aneuploid cells was 8.1% in the larval brains of diploid offspring; 10.4% in triploid offspring; and 12.6% in tetraploid offspring (Fig. 3E). There is a strong correlation between the increase in ploidy with the proportion of aneuploid cells in the larval brain tissue of the engineered parthenogens (*r* = 0.9999, *p* = 7.8 × 10^−3^). The distribution of chromosomes that were lost in parthenogenetic *D. melanogaster* was more evenly spread (Fig. 3F). We did not observe any clustering of mitotic cells exhibiting aneuploidy, suggesting that this is not the result of a clonal event being propagated (Fig. 3G). However, similar to the findings in larval brains of *D. mercatorum* parthenogens, the nuclei in all larval brain cells of diploid parthenogenetic females appeared larger and less densely packed. Elevated Myc is known to result in enlarged cells in diploid tissues of *Drosophila* (Grewal et al. 2005), and since *Myc* expression is high in both *D. mercatorum* and *D. melanogaster* parthenogens, we propose that this may be the cause of the increased cell size in both species. However, it is also possible that neuroblast enlargement is a consequence of the concurrent aneuploidy that is taking place. Why this does not occur in the sexually reproduced animals of the same genetic background is not clear, although we speculate that there is an additional, and as of yet unidentified, factor that stochastically changes gene expression during parthenogenesis that contributes to this process.

**Fig. 4:**
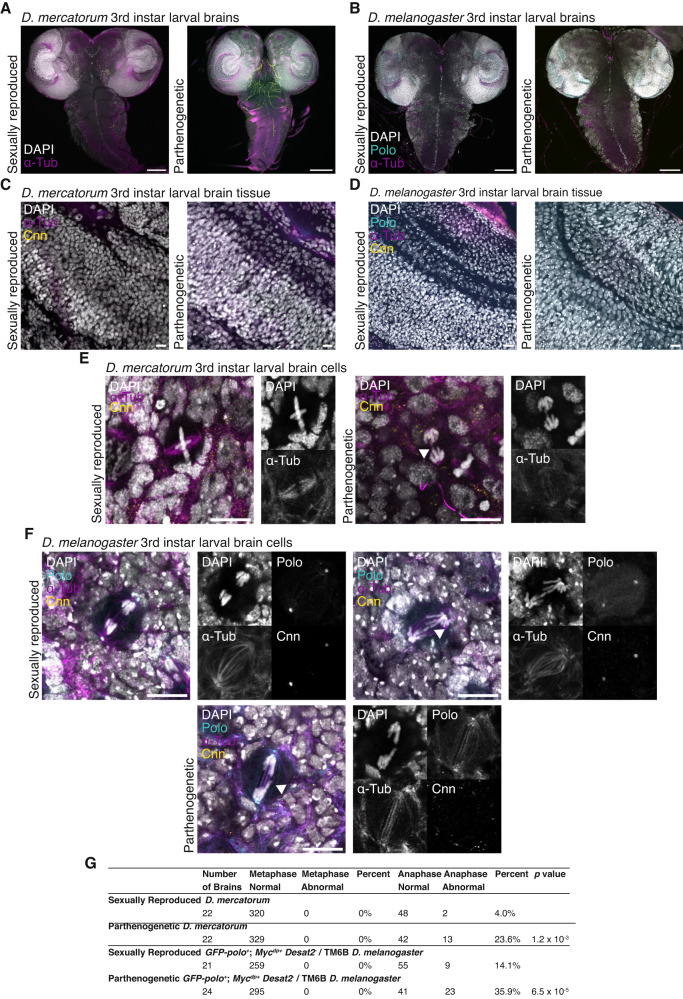
Sexually reproducing and parthenogenetic *D. melanogaster* and *D. mercatorum* brains. **A** Whole brains of sexually and parthenogenetically reproduced *D. mercatorum* 3rd instar larvae brains. **B** Whole brains of sexually and parthenogenetically reproduced *GFP-polo*^*+*^*; Myc*^*dp+*^ *Desat2*^*-*^/TM6B *D. melanogaster* 3rd instar larvae brains. **A**, **B** DAPI/DNA (white), GFP-polo (cyan), Phalloidin (magenta), and Asterless/Asl (yellow). Scale bar, 100 μm. **C** Brain lobes of sexually and parthenogenetically reproduced *D. mercatorum* 3rd instar larvae brains. **D** Brain lobes of sexually and parthenogenetically reproduced *GFP-polo*^*+*^*; Myc*^*dp+*^ *Desat2*^*-*^/TM6B *D. melanogaster* 3rd instar larvae brains. **E** Metaphase and anaphase cells in sexually and parthenogenetically reproduced *D. mercatorum* 3rd instar larvae brains. Arrowhead indicating lagging chromosomes. **F** Anaphase cells in sexually and parthenogenetically reproduced *GFP-polo*^*+*^*; Myc*^*dp+*^ *Desat2*^*−*^/TM6B *D. melanogaster* 3rd instar larvae brains. Arrowhead indicating lagging chromosomes. **C**–**F** DAPI/DNA (white), GFP-polo (cyan), α-Tubulin (magenta), and Cnn (yellow). Scale bar, 10 μm. **G** Table shows phenotypes of mitotic cells that were in metaphase or anaphase for both *D. mercatorum* and *GFP-polo*^*+*^*; Myc*^*dp+*^ *Desat2*^*-*^/TM6B *D. melanogaster* 3rd instar larvae brains. The *p* value was calculated with Fisher’s exact test.

The prevalence of aneuploidy led us to carry out a more detailed exanimation of the larval brains of both the sexually reproducing and parthenogenetic *D. mercatorum* and the sexually reproducing and parthenogenetic *GFP-polo*^*+*^; *Myc*^*+dp*^ *Desat2*^*-*^/TM6B *D. melanogaster*. In *D. mercatorum* and *GFP-polo*^*+*^; *Myc*^*+dp*^ *Desat2*^*-*^/TM6B *D. melanogaster* there was no apparent sign of dysplastic or abnormal tissue in either sexually reproduced or parthenogenetic offspring (Fig. 4A, B). The larval brains appeared healthy and exhibited overall similar morphological characteristics between larvae produced by the two reproductive modes within the same strain. Hence, despite the presence of aneuploidy within the larval brain tissue, it does not seem to have a negative impact on the overall morphology. However, all cells within the tissue appeared more densely packed in the sexually reproduced offspring compared to the parthenogenetic offspring from both species (Fig. 4C, D), confirming the results of the karyotype preparations (Figs. 2F and 3G). Mitotic cells in sexually and parthenogenetically reproduced *D. mercatorum* and genetically engineered *GFP-polo*^*+*^; *Myc*^*+dp*^ *Desat2*^*-*^/TM6B *D. melanogaster* larval brain tissue all appeared normal when in metaphase, regardless of reproductive mode. However, we observed that there were lagging chromosomes or anaphase bridges present in 27.1% of anaphase cells in *D. mercatorum* parthenogens compared to 4.2% in the sexually preproduced offspring and 35.9% in *D. melanogaster* parthenogens compared to 14.1% in the sexually reproduced offspring (Fig. 4E–G). It seems therefore that chromosome nondisjunction is the likely cause of aneuploidy in both *D. mercatorum* and genetically engineered *GFP-polo*^*+*^; *Myc*^*+dp*^ *Desat2*^*-*^/TM6B *D. melanogaster* parthenogens.

## Discussion

In this study, we provide the first characterization of the incidence of aneuploidy in the larval brains of a naturally occurring facultative parthenogenetic strain of *D. mercatorum* and a genetically engineered facultative parthenogenetic strain of *D. melanogaster*. Our findings reveal a stark contrast in the extent of aneuploidy between parthenogenetic offspring and sexually reproduced animals within the same strain. These observations strongly suggest that sexual reproduction offers a protective mechanism for safeguarding genomic integrity and prevent the occurrence of aneuploidy in a genetic background that favors the occurrence of these abnormalities. In contrast, the parthenogenetic offspring have greater intra-individual genomic variability than their sexually reproduced counterparts. Studies in budding yeast have demonstrated that certain phenotypic effects arising from aneuploidy in haploid strains can be mitigated by an increase in ploidy levels (Oromendia et al. 2012; Santaguida and Amon 2015; Torres et al. 2007). This suggests that elevated ploidy levels alleviate the stress associated with aneuploidy. In our study, we also observe a positive correlation between increased polyploidy and higher levels of aneuploidy in *D. melanogaster*. This implies that aneuploid cells may have a greater propensity for survival when accompanied by polyploidy or that polyploidy itself predisposes cells to an elevated incidence of aneuploidy in certain animals. The preferential loss of certain chromosomes and the absence of apparent detrimental effects on larval brain tissue suggests that *Drosophila* parthenogens have mechanisms to tolerate and compensate for aneuploidy.

The four main causes of aneuploidy (described in Sansregret and Swanton 2017; Siegel and Amon 2012) are SAC failure, centrosome amplification, microtubule defects, and sister chromatid adhesion defects. Aneuploidy within parthenogenetic *D. melanogaster* is likely a consequence of the combination of genes involved in parthenogenesis; elevated *Myc*, or *Polo* and decreased *Desat2*. Elevated *Myc* expression has been associated with aneuploidy in humans through a variety of mechanisms (Jones et al. 2010; Sansregret and Swanton 2017; Weaver et al. 1999), most notably by altering the expression of genes involved in centrosome amplification and sister chromatid adhesion defects (Duijf and Benezra 2013; Sansregret and Swanton 2017; Zeng et al. 2010). Parthenogenetic *D. mercatorum* exhibited greater expression of multiple centrosome genes (Sperling et al. 2023), and parthenogenetic embryos displayed the formation of multiple MTOCs (Riparbelli and Callaini 2003). However, as development proceeds cells retain only the expected two centrosomes as is seen in the developing embryo (Sperling et al. 2023). Thus, centrosome amplification is not a cause of aneuploidy at these late stages of development. The cause is likely to be in sister chromatid adhesion because we observed germline nondisjunction and anaphase bridges during mitosis. Therefore, we propose that a culprit causing the increased incidence of aneuploidy in both *D. melanogaster* and *D. mercatorum* larval brain tissue may be altered *Myc* expression resulting in sister chromosome cohesion defects.

The genome heterogeneity observed in somatic tissues of parthenogenetic *Drosophila* carries significant implications, as it could provide these animals with genetic variation. The presence of high intra-individual genetic variability in parthenogenetic populations suggests that these populations may possess a capacity for adaptation, which could explain the selection for an increase in facultative parthenogenesis over generations or the genetic diversity that has been observed in some parthenogenetic populations (Niklasson et al. 2004; Stalker 1956). This phenomenon raises intriguing possibilities for the evolution of parthenogenesis if there were continued selection pressure for parthenogenesis in sexually reproducing populations combined with the occurrence of aneuploidy and subsequent DNA damage, may contribute to the emergence of enhanced parthenogenetic reproductive ability. Understanding the occurrence of aneuploidy in natural populations of parthenogenetic *D. mercatorum* and other organisms presents an exciting avenue for future research. Investigating the evolutionary implications and mechanisms underlying aneuploidy and its relationship to parthenogenesis could provide valuable insights into the dynamics of reproduction and adaptation in these species.

In obligate parthenogens, there have been many documented cases of aneuploidy (Sperling and Glover 2023a), including both germline and somatically derived aneuploidy in many species of obligately parthenogenetic aphids and nematodes (Blackman 1980; Castagnone-Sereno 2006). There are also incidences of aneuploidy in obligately parthenogenetic flies that is likely a consequence of somatic or sex chromosome elimination (Sperling and Glover 2023a). When examining the nematodes, it does not appear that aneuploidy is tied specifically to apomixis (bypassing meiosis) or automixis (meiosis is fully or partially retained), since it occurs in both (Castagnone-Sereno 2006). It also occurs in the facultative parthenogens in our study and the above mentioned obligate parthenogens, therefore it is also not linked to the type of parthenogenesis. Therefore, aneuploidy may be tied to the molecular mechanism of parthenogenesis which will only become apparent once we know the cause of parthenogenesis in a broader range of animals.

While aneuploidy may confer certain benefits in the context of parthenogenesis, it is also likely to come with costs to the development of the animal. In the facultative parthenogens we studied it is apparent that the careful regulation of mitosis is not regained after the initiation of parthenogenesis. Therefore, the paradox of needing the regain control of mitosis does appear to happen in *Drosophila*, explaining the low level of adult offspring produced by facultative parthenogenetic *Drosophila* (Eisman and Kaufman 2007; Kramer et al. 2002). Multiple developmental abnormalities have been documented in various facultative parthenogenetic *Drosophila* species (Carson 1961; Kramer et al. 2002; Stalker 1954). Examples include the development of abnormal numbers of legs, abdominal abnormalities, and the presence of the Minute phenotype in *D. parthenogenetica* (Stalker 1954). The pleiotropic nature of these developmental abnormalities makes it difficult to pinpoint their specific causes, but all could be a consequence of genome instability. It is possible that these defects lead to developmental delays as the organism attempts to resolve the errors and achieve normal adulthood, similar to observations in birds (Ramachandran and McDaniel 2018). Hence, the genetic and genomic changes enabling aneuploidy in facultative parthenogenesis may cause developmental delays and abnormalities that may be detrimental to the survival of most offspring produced through this reproductive mode. Our findings provide genetic and mechanistic insights into the potential drawbacks of facultative parthenogenesis, shedding light onto why this mode of reproduction may not be entirely beneficial from a developmental perspective. The transition to facultative parthenogenesis appears to involve a delicate balance, encompassing both potential benefits and developmental costs.

The prevalence of facultative parthenogenesis in the *Drosophila* genus, where 76% of species have the capacity for some level of parthenogenetic reproduction, and midges and mosquitoes suggests that similar phenomena may occur in other dipteran species (Sperling and Glover 2023a). Consequently, our findings are likely not limited to *Drosophila* alone but have broader relevance in understanding the evolutionary mechanisms underlying parthenogenetic reproductive strategies and the role of chromosome instability in shaping genetic diversity across various organisms. Further exploration of these phenomena in diverse organisms will enhance our understanding of the adaptive significance and evolutionary consequences of facultative parthenogenesis and aneuploidy, thereby advancing our knowledge of reproductive strategies and genetic diversity across the animal kingdom.

### Supplementary information

Supplemental Table 1

## References

[CR1] Ashburner M (1989). Drosophila: a laboratory handbook. *Cold Spring Harbor Laboratory Press, Cold Spring Harbor, NY*

[CR2] Basto R, Lau J, Vinogradova T, Gardiol A, Woods CG, Khodjakov A (2006). Flies without centrioles. Cell.

[CR3] Blackman RL (1980). Chromosome numbers in the Aphididae and their taxonomic significance. Syst Entomol.

[CR4] Carson HL (1961). Parthenogenesis in *Drosophila robusta*. Am Nat.

[CR5] Castagnone-Sereno P (2006). Genetic variability and adaptive evolution in parthenogenetic root-knot nematodes. Heredity.

[CR6] Dang CV (2012). MYC on the path to cancer. Cell.

[CR7] DeSalle R, Slightom J, Zimmer E (1986). The molecular through ecological genetics of *abnormal abdomen*. II. Ribosomal DNA polymorphism is associated with the abnormal abdomen syndrome in *Drosophila mercatorum*. Genetics.

[CR8] Duijf PH, Benezra R (2013). The cancer biology of whole-chromosome instability. Oncogene.

[CR9] Eisman RC, Kaufman TC (2007). Cytological investigation of the mechanism of parthenogenesis in *Drosophila mercatorum*. Fly.

[CR10] Engelstadter J (2008). Constraints on the evolution of asexual reproduction. BioEssays N Rev Mol Cell Dev Biol.

[CR11] Greenberg AJ, Moran JR, Coyne JA, Wu C-I (2003). Ecological adaptation during incipient speciation revealed by precise gene replacement. Science.

[CR12] Grewal SS, Li L, Orian A, Eisenman RN, Edgar BA (2005). Myc-dependent regulation of ribosomal RNA synthesis during Drosophila development. Nat Cell Biol.

[CR13] Grifoni D, Bellosta P (2015). Drosophila Myc: a master regulator of cellular performance. Biochim Biophys Acta.

[CR14] Jones L, Wei G, Sevcikova S, Phan V, Jain S, Shieh A (2010). Gain of MYC underlies recurrent trisomy of the MYC chromosome in acute promyelocytic leukemia. J Exp Med.

[CR15] Kramer MG, Templeton AR, Miller KG (2002). Evolutionary implications of developmental instability in parthenogenetic *Drosophila mercatorum*. I. Comparison of several strains with different genotypes. Evol Dev.

[CR16] Markow TA (2013). Parents without partners: Drosophila as a Model for Understanding the Mechanisms and Evolution of Parthenogenesis. G3.

[CR17] Muller HJ (1940) Bearings of the ‘Drosophila’ work on systematics. *The new systematics edited by Huxley J Clarendon Press, Oxford***:** p 185–268

[CR18] Niklasson M, Tomiuk J, Parker ED (2004). Maintenance of clonal diversity in Dipsa bifurcata (Fallen, 1810) (Diptera: Lonchopteridae). I. Fluctuating seasonal selection moulds long-term coexistence. Heredity.

[CR19] Oromendia AB, Dodgson SE, Amon A (2012). Aneuploidy causes proteotoxic stress in yeast. Genes Dev.

[CR20] Ramachandran R, McDaniel CD (2018). Parthenogenesis in birds: a review. Reproduction.

[CR21] Rehen SK, McConnell MJ, Kaushal D, Kingsbury MA, Yang AH, Chun J (2001). Chromosomal variation in neurons of the developing and adult mammalian nervous system. PNAS.

[CR22] Rehen SK, Yung YC, McCreight MP, Kaushal D, Yang AH, Almeida BS (2005). Constitutional aneuploidy in the normal human brain. J Neurosci.

[CR23] Riparbelli MG, Callaini G (2003). Drosophila parthenogenesis: a model for de novo centrosome assembly. Dev Biol.

[CR24] Sansregret L, Swanton C (2017) The role of aneuploidy in cancer evolution. *Cold Spring Harb Perspect Med* 7(1) 10.1101/cshperspect.a028373 PMC5204330 28049655

[CR25] Santaguida S, Amon A (2015). Short- and long-term effects of chromosome mis-segregation and aneuploidy. Nat Rev Mol cell Biol.

[CR26] Schaeffer SW (2018). Muller “elements” in Drosophila: how the search for the genetic basis for speciation led to the birth of comparative genomics. Genetics.

[CR27] Schindelin J, Arganda-Carreras I, Frise E, Kaynig V, Longair M, Pietzsch T (2012). Fiji: an open-source platform for biological-image analysis. Nat Methods.

[CR28] Siegel JJ, Amon A (2012). New insights into the troubles of aneuploidy. Annu Rev Cell Dev Biol.

[CR29] Sperling AL, Fabian DK, Garrison EK, Glover DM (2023). A genetic basis for facultative parthenogenesis in Drosophila. Curr Biol.

[CR30] Sperling AL, Glover DM (2023a) Parthenogenesis in dipterans: a genetic perspective. *Proc R Soc B: Biol Sci* 290. 10.1098/rspb.2023.0261 PMC10031431 36946111

[CR31] Sperling AL, Glover DM (2023). Protocol for screening facultative parthenogenesis in Drosophila. STAR Protoc.

[CR32] Stalker HD (1954). Parthenogenesis in Drosophila. Genetics.

[CR33] Stalker HD (1956). On the Evolution of Parthenogenesis in *Lonchoptera* (*Diptera*). Evolution.

[CR34] Suomalainen E (1950) Parthenogenesis in animals. *Adv Genet*, p 19323 15425392

[CR35] Suvorov A, Kim BY, Wang J, Armstrong EE, Peede D, D’Agostino ERR (2022). Widespread introgression across a phylogeny of 155 Drosophila genomes. Curr Biol CB.

[CR36] Takahashi A, Tsaur S-C, Coyne JA, Wu C-I (2001). The nucleotide changes governing cuticular hydrocarbon variation and their evolution in *Drosophila melanogaster*. PNAS.

[CR37] Takai N, Hamanaka R, Yoshimatsu J, Miyakawa I (2005). Polo-like kinases (Plks) and cancer. Oncogene.

[CR38] Torres EM, Sokolsky T, Tucker CM, Chan LY, Boselli M, Dunham MJ (2007). Effects of aneuploidy on cellular physiology and cell division in haploid yeast. Science.

[CR39] Weaver ZA, McCormack SJ, Liyanage M, du Manoir S, Coleman A, Schröck E (1999). A recurring pattern of chromosomal aberrations in mammary gland tumors of MMTV-cmyc transgenic mice. Genes, Chromosomes Cancer.

[CR40] Whiting JH, Pliley MD, Farmer JL, Jeffery DE (1989). In situ hybridization analysis of chromosomal homologies in *Drosophila melanogaster* and *Drosophila virilis*. Genetics.

[CR41] Yurov YB, Iourov IY, Monakhov VV, Soloviev IV, Vostrikov VM, Vorsanova SG (2005). The variation of aneuploidy frequency in the developing and adult human brain revealed by an interphase FISH study. J Histochem Cytochem.

[CR42] Yurov YB, Iourov IY, Vorsanova SG, Liehr T, Kolotii AD, Kutsev SI (2007). Aneuploidy and confined chromosomal mosaicism in the developing human brain. PloS one.

[CR43] Zeng X, Shaikh FY, Harrison MK, Adon AM, Trimboli AJ, Carroll KA (2010). The Ras oncogene signals centrosome amplification in mammary epithelial cells through cyclin D1/Cdk4 and Nek2. Oncogene.

