# Supplementary material for: Aneuploidy during development in facultative parthenogenetic Drosophila
Source: Heredity (Edinb). 2023 Nov 28;132(2):89–97. doi: 10.1038/s41437-023-00664-z (PMC10844303; doi:10.1038/s41437-023-00664-z)
Supplement: Supplementary file 1 — Supplemental Table 1 [file 41437_2023_664_MOESM1_ESM.pdf]

A

D. mercatorum

Individual	Sexually Reproducing			Parthenogenetic		
	Euploid	Aneuploid	percent	Euploid	Aneuploid	percent
1	7	0	**0%**	15	0	**0%**
2	4	0	**0%**	16	0	**0%**
3	19	0	**0%**	3	1	**25.0%**
4	15	1	**6.3%**	11	0	**0%**
5	13	0	**0%**	19	4	**17.4%**
6	15	0	**0%**	17	4	**19.0%**
7	11	4	**26.7%**	32	11	**25.6%**
8	14	0	**0%**	51	5	**8.9%**
9	3	1	**25.0%**	17	0	**0%**
10	11	0	**0%**	44	6	**12.0%**
11	35	0	**0%**	22	8	**26.7%**
12	15	3	**16.7%**	41	8	**16.3%**
13	20	1	**4.8%**	40	4	**9.1%**
14	26	1	**3.7%**	27	0	**0%**
15	21	0	**0%**	43	10	**18.9%**
16	36	2	**5.0%**	21	2	**8.7%**
17	38	0	**0%**	57	5	8.1%
18	24	0	**0%**	33	2	**5.7%**
19	30	2	**6.3%**	16	1	**5.9%**
20	46	2	**4.2%**	18	0	**0%**
21	28	1	**3.4%**	20	0	**0%**
22	31	1	**3.1%**	22	1	**4.3%**
23	4	0	**0%**	32	1	**3.0%**
24	10	0	**0%**	39	2	**4.9%**
25	17	0	**0%**	15	3	**16.7%**
26	7	0	**0%**	29	3	**9.4%**
27	3	0	**0%**	28	1	**3.4%**
28	11	0	**0%**	31	4	**11.4%**
29	8	0	**0%**	23	5	**17.9%**
30	18	1	**5.3%**	27	2	**6.9%**
Total	540	20	**3.6%**	809	93	**10.3%**

B

D. melanogaster GFP-polo^{+}; Myc^{dp+} Desat2^{-} / TM6B

Individual	Sexually Reproducing			Parthenogenetic		
	Euploid	Aneuploid	percent	Euploid	Aneuploid	percent
1	54	0	**0%**	57	3	**5.0%**
2	50	2	**3.8%**	41	4	**8.9%**
3	46	0	**0%**	54	1	**1.8%**
4	53	1	**1.9%**	26	1	**3.7%**
5	50	1	**2.0%**	7	4	**36.4%**
6	59	5	**7.8%**	46	4	**8.0%**
7	28	1	**3.4%**	40	4	**9.1%**
8	20	0	**0%**	45	1	**2.2%**
9	41	2	**4.7%**	42	8	**16.0%**
10	31	2	**6.1%**	39	11	**22.0%**
11	53	1	**1.9%**	52	0	**0%**
12	52	2	**3.7%**	41	7	**14.6%**
13	47	1	**2.1%**	46	3	**6.1%**
14	52	2	**3.7%**	34	1	**2.9%**
15	44	0	**0%**	3	2	**40.0%**
16	76	2	**2.6%**	17	6	**26.1%**
17	25	4	**13.8%**	5	3	**37.3%**
18	44	5	**10.2%**	67	13	**16.3%**
19	25	0	**0%**	42	5	**10.6%**
20	17	2	**10.5%**	44	4	**8.3%**
21	48	0	**0%**	18	1	**5.3%**
22	50	2	**3.8%**	13	1	**7.1%**
23	34	0	**0%**	34	1	**2.9%**
24	18	0	**0%**	41	1	**2.4%**
25	50	2	**3.7%**	34	4	**10.5%**
26	42	1	**2.3%**	26	10	**27.8%**
27	50	1	**2.0%**	38	3	**7.3%**
28	36	1	**2.7%**	34	1	**2.9%**
29	27	0	**0%**	26	3	**10.3%**
30	26	0	**0%**	44	3	**6.4%**
Total	1248	40	**3.1%**	1056	113	**9.7%**

Table S1: Sexually reproducing and parthenogenetic *D. mercatorum* and *GFP-polo^{+}*; *Myc^{dp+} Desat2^{-}* / TM6B 3rd *D. melanogaste*r aneuploidy in instar larvae brains, summarized in Figures 2D and 3E.
